# Commentary: Variant Signal Peptides of Vaccine Antigen, FHbp, Impair Processing Affecting Surface Localization and Antibody-Mediated Killing in Most Meningococcal Isolates

**DOI:** 10.3389/fmicb.2020.538209

**Published:** 2020-11-06

**Authors:** Paul Liberator, Robert G. K. Donald, Paul Balmer, Jamie Findlow, Annaliesa S. Anderson

**Affiliations:** ^1^Pfizer Vaccine Research and Development, Pearl River, NY, United States; ^2^Pfizer Vaccines Medical Development, Scientific and Clinical Affairs, Collegeville, PA, United States; ^3^Pfizer Medical Development, Scientific and Clinical Affairs, Tadworth, United Kingdom

**Keywords:** signal peptide, vaccine, FHbp, serogroup B, *Neisseria meningitidis*, Trumenba®, Bexsero®, antibody-mediated killing

## Introduction

Meningitis and/or septicemia caused by *Neisseria meningitidis* remains a serious public health concern. The factor H binding protein (FHbp) is a surface lipoprotein and an antigen component of the two vaccines that are licensed in the US for the prevention of *N. meningitidis* serogroup B (MenB) disease (TRUMENBA®, 2014; BEXSERO®, 2015). Both vaccines were licensed based on the demonstration that they induce antibody responses that kill MenB isolates in serum bactericidal activity assays using human complement (hSBA). hSBA titers of >1:4 are the established correlate of protection against meningococcal disease (Goldschneider et al., 1969).

## Conclusions From da Silva et al. (2019)

Four classes of FHbp signal peptide (SP) sequences (SP1-SP4) were identified. After screening 18 MenB strains, the authors provide data illustrating that FHbp variants coding for classes SP2-SP4 are not exported to the cell surface via the traditional lipoprotein pathway. These FHbp variants could still be detected on the cell surface (albeit at levels lower than SP1 FHbp variants) and were able to bind the cognate ligand, factor H. FHbp-specific monoclonal antibodies were used to conclude that strains expressing SP2-4 FHbp variants were less susceptible in hSBAs than strains expressing SP1 FHbp variants (which utilize the traditional lipoprotein transport mechanism). The authors note that SP1 FHbp variants are representative of <12% of invasive MenB isolates collected in the UK during 2009-2017 and caution that statements regarding the breadth of coverage for Trumenba® need to consider the SP sequence of FHbp variants expressed by target strains used to demonstrate the immune response to vaccination.

## Rebuttal

da Silva et al. (2019) suggest that Trumenba® coverage (and to a lesser extent Bexsero®) may be overstated considering their findings. The immune response to Trumenba® was defined during clinical development using strains expressing FHbp variants that: (i) were heterologous to vaccine antigens, (ii) were representative of prevalent variants, (iii) express antigen at representative levels, and (iv) were selected in an unbiased manner (Donald et al., 2017; Harris et al., 2017). A considerable body of data was used to support vaccine licensure by national regulatory authorities. In the European Summary of Product Characteristics, the data for Trumenba® highlight the potential to kill over 91% of isolates (https://www.ema.europa.eu/en/documents/product-information/trumenba-epar-product-information_en.pdf) and 78% for Bexsero® (https://www.ema.europa.eu/en/documents/product-information/bexsero-epar-product-information_en.pdf). Licensure in both instances was supported by assays that determined the level of FHbp expression using thousands of MenB strains collected systematically from different geographical regions so that the breadth of coverage could be ascertained. These data were generated prior to the recognition of alternative FHbp maturation pathways that are experimentally detailed in the da Silva et al. (2019) publication. In the US, Trumenba® licensure was based on demonstrating the ability of vaccine induced antibodies to kill a broad range of MenB strains with diverse FHbp variants and as such is the only vaccine approved for broad coverage against diverse MenB strains. The authors suggest that their findings translate to the potential for ineffective coverage elicited by Trumenba®. We are concerned that this misleading statement will impact discussions between medical professionals and their patients regarding decisions to vaccinate. For this reason, we are providing details of the MenB strains that were used to demonstrate the breadth of coverage of Trumenba®.

Assessments of the breadth of coverage to vaccination with Trumenba® in phase 3 clinical trials used four primary hSBA strains and ten additional test strains that met the requirements described earlier. Characteristics of these strains are listed in Table 1. SP sequences corresponding to each of the four classes of SP allelic variants are represented multiple times. Therefore, regardless of the mechanism of biochemical processing, these clinically relevant hSBA strains each express surface exposed FHbp and are susceptible to Trumenba® immune sera (Harris et al., 2017; Ostergaard et al., 2017; Taha et al., 2017). Thus, the concerns raised in da Silva et al. (2019) regarding the potential coverage of Trumenba® can now be shown to be unfounded. Likewise, the proposed algorithm using SP sequences to predict whether an isolate expresses FHbp at levels sufficient to be susceptible to the bactericidal activity of Trumenba® immune sera would also not be relevant based on the results we present here.

**Table 1:** Characteristics of primary and additional hSBA strains used to determine the immune response to Trumenba®.

**Strain ID**	**Clonal complex**	**FHbp variant**	**GenBank accession number**	**Signal peptide class**	**MEASURE MFI (±1 SD)^**a**^**	**% Adolescent subjects (10–18 yr) with hSBA titer ≥prespecified endpoints (95% CI)^**b**^**	**hSBA GMTs for adolescents (10–18 yr) (95% CI)^**b**^**
**Primary hSBA strains**							
PMB80	41/44	A22	MT013142	3	3127 (2440, 4007)	97.7 (96.7, 98.4)	85.6 (81.27, 90.10)
PMB2001	213	A56	MT013143	1	5002 (3903, 6410)	99.5 (99.0, 99.8)	218.4 (206.63, 230.88)
PMB2948	32	B24	MT013145	1	6967 (5436, 8929)	86.4 (84.5, 88.2)	23.7 (22.35, 25.04)
PMB2707	269	B44	MT013144	4	11283 (8804, 14461)	88.6 (86.8, 90.3)	49.3 (45.63, 53.34)
**Additional hSBA strains**							
PMB3175	32	A29	MT013155	4	3839 (2995, 4920)	98.7 (96.6, 99.6)	93.6 (85.19, 102.90)
PMB3010	461	A06	MT013148	4	3370 (2629, 4319)	95.6 (92.6, 97.6)	78.7 (71.20, 86.99)
PMB3040	162	A07	MT013150	3	1379 (1076, 1767)	96.6 (93.9, 98.4)	64.8 (59.27, 70.75)
PMB824	35	A12	MT013149	3	2540 (1982, 3255)	75.3 (70.0, 80.2)	22.3 (20.38, 24.31)
PMB1672	103	A15	MT013146	3	2995 (2337, 3838)	86.3 (81.8, 90.1)	30.5 (27.01, 34.40)
PMB1989	8	A19	MT013147	3	1934 (1509, 2478)	92.2 (88.5, 95.0)	57.0 (50.93, 63.79)
PMB1256	41/44	B03	MT013151	2	3976 (3102, 5095)	92.2 (88.5, 95.0)	50.6 (44.55, 57.46)
PMB866	269	B09	MT013152	2	2089 (1630, 2677)	86.3 (81.9, 90.1)	22.8 (20.48, 25.42)
PMB431	41/44	B15	MT013153	1	3785 (2953, 4851)	98.3 (96.1, 99.5)	47.0 (43.17, 51.22)
PMB648	41/44	B16	MT013154	3	2347 (1831, 3008)	80.1 (75.1, 84.5)	21.4 (18.98, 24.10)

a *Harris et al. (2017) and McNeil et al. (2018)*.

b *Ostergaard et al. (2017)*.

*CI, confidence interval; FHbp, factor H binding protein; GMT, geometric mean titer; hSBA, serum bactericidal assay using human complement; ID, strain identifier; MFI, mean fluorescence intensity; SD, standard deviation*.

The da Silva et al. (2019) manuscript also cites a report which concluded that FHbp expressed at levels of at least 757 molecules per cell (~135 pg FHbp/μg of cellular extract) is required for killing by FHbp-specific antibodies derived from mice immunized with Bexsero® (Biagini et al., 2016). Pfizer has demonstrated that strains expressing lower levels of FHbp (<1,000 mean fluorescence intensity, or the equivalent <30 pg FHbp/μg of cellular extract) can be killed in hSBAs using Trumenba® clinical immune sera (McNeil et al., 2018). The difference in the FHbp threshold level sufficient for killing may be attributed to differences in the bactericidal responses elicited by the vaccines or the reagents and methods used, including the source of immune sera (mouse for Bexsero®, human for Trumenba®) or the source of complement (rabbit and rSBAs for Bexsero®, human and hSBAs for Trumenba®). Nonetheless, both sets of results should be considered to provide a balanced hypothesis on the level of expression required for FHbp-specific antibody-mediated killing. Indeed, the basis for many of the conclusions in da Silva et al. (2019) draw upon non-clinical data obtained for Bexsero®.

## Conclusion

The experimental findings reported by da Silva et al. (2019) clearly demonstrate that post-translational processing and surface localization of FHbp are complex, challenging traditional mechanisms of lipoprotein maturation in bacteria. Though the results reported are mechanistically interesting, the proposed impact of these findings on the breadth of coverage of Trumenba®, a vaccine licensed to prevent MenB infections, has not taken into account the hSBA data that was used to support licensure of Trumenba®. The results presented herein illustrate that MenB strains that code for each of the four SP alleles express FHbp at the bacterial surface and are susceptible to complement-mediated killing by antibodies elicited by Trumenba®. The data summarized in this Commentary address the concerns raised in da Silva et al. (2019) and highlight the potential for Trumenba® to prevent meningococcal disease caused by diverse MenB strains.

## Author Contributions

All authors listed have made a substantial, direct and intellectual contribution to the work, and approved it for publication.

## Conflict of Interest

PL, RD, PB, JF, and AA are Pfizer employees.
